# Abundance, rather than composition, of methane‐cycling microbes mainly affects methane emissions from different vegetation soils in the Zoige alpine wetland

**DOI:** 10.1002/mbo3.699

**Published:** 2018-07-26

**Authors:** Yanfen Zhang, Mengmeng Cui, Jingbo Duan, Xuliang Zhuang, Guoqiang Zhuang, Anzhou Ma

**Affiliations:** ^1^ Key Laboratory of Environmental Biotechnology Research Center for Eco‐Environmental Sciences Chinese Academy of Sciences Beijing China; ^2^ University of Chinese Academy of Sciences Beijing China; ^3^ National Laboratory of Biomacromolecules Institute of Biophysics Chinese Academy of Sciences Beijing China; ^4^ Key Laboratory of Environmental Optics & Technology Anhui Institute of Optics and Fine Mechanics Chinese Academy of Sciences Hefei China

**Keywords:** abundance, methane flux, methanogens, methanotrophs, vegetation species

## Abstract

Methane fluxes, which are controlled by methanogens and methanotrophs, vary among wetland vegetation species. In this study, we investigated belowground methanogens and methanotrophs in two soils under two different dominant vegetation species with different methane fluxes in the Zoige wetland, which was slightly but significantly (*p* ≤ 0.05) higher in soils covered by *Carex muliensis* than that in soils covered by *Eleocharis valleculosa*. Real‐time quantitative PCR and Illumina MiSeq sequencing methods were used to elucidate the microbial communities based on the key genes involved in methane production and oxidation. The absolute abundances of methanogens and methanotrophs of samples from *C*.* muliensis* were 1.80 ± 0.07 × 10^6^ and 4.03 ± 0.28 × 10^6^ copies g‐soil^−1^, respectively, and which from *E. valleculosa* were 3.99 ± 0.19 × 10^5^ and 2.53 ± 0.22 × 10^6^ copies g‐soil^−1^ , respectively. The *t*‐test result showed that both the abundance of methanogens and methanotrophs from *C. muliensis* were significantly higher (*p* ≤ 0.05) than that of samples from *E. valleculosa*. However, the diversities and compositions of both methanogens and methanotrophs showed no significant differences (*p* ≥ 0.05) between vegetation species. The path analysis showed that the microbial abundance had a greater effect than the microbial diversity on methane production potentials and the regression analysis also showed that the methane emissions significantly (*p* ≤ 0.05) varied with the abundance of methane‐cycling microbes. These findings imply that abundance rather than diversity and composition of a methane‐cycling microbial community is the major contributor to the variations in methane emissions between vegetation types in the Zoige wetland.

## 1 INTRODUCTION

As an important greenhouse gas, methane emissions have been estimated to be responsible for approximately 20% of Earth's warming since preindustrial times (Kirschke et al., 2013). An analysis of sources and sinks of methane has shown that natural wetlands are the largest global natural methane sources (Aronson, Allison, & Helliker, 2013). Methane emissions from permafrost wetlands are of particular concern as they strongly contribute to carbon sequestration, and permafrost wetlands contain approximately one‐third of the global soil carbon. Moreover, permafrost wetlands make up to 50% of the total global wetland area (Lehner & DöLL, 2004). The warming climate therefore necessitates the study of methane in these wetlands.

Many ecosystems are affected by the increasingly warming climate, including wetlands. In wetlands, the most obvious change under climatic warming is the composition change in vegetation communities (Britton, Hewison, Mitchell, & Riach, 2017). In addition, a vegetation community can change even with air temperature variations of 1–2°C in alpine areas (Cannone, Sgorbati, & Guglielmin, 2007). Under composition changes, some species are lost (Klein, Harte, & Zhao, 2004), and some species are replaced (Wang, Li, Huang, & Li, 2005). The variations in vegetation species can affect the emissions of greenhouse gases, including methane. Moreover, the differences in methane fluxes among different vegetation types have often been reported in wetlands (Bhullar, Iravani, Edwards, & Venterink, 2013; Chen, Wu, Wang, Gao, & Peng, 2011; Cui et al., 2015; Joabsson & Christensen, 2001; Ström, Ekberg, Mastepanov, & Røjle Christensen, 2003; Ström, Mastepanov, & Christensen, 2005).

The Zoige wetland is located on the Tibetan Plateau, which is the largest high‐altitude and low‐latitude permafrost area on Earth (Cheng, 2005) and is sensitive to global changes (Qiu, 2008). This wetland is also a major methane emission hotspot (Tomoko, 1999) with large carbon stocks, which accounts for 6.2% of the organic carbon storage in China (Gao, Ou, Zhang, & Zhang, 2007). The vegetation cover in the Zoige wetland is mainly *Carex muliensis* (Cm) and *Eleocharis valleculosa* (Ev), which both have high methane emission rates (Chen, Yao, et al., 2008). Moreover, methane fluxes have been shown to vary between the two vegetation species, with higher methane emissions from Cm (Chen et al., 2009).

Methane fluxes are actually controlled through production and consumption by the microorganisms at the surface or in the vicinity of roots (Conrad, 2004). The microbes that are responsible for producing methane are called methanogens, and the consumers are called methanotrophs. Research on the methanogens and methanotrophs underneath different vegetation types can provide a more detailed explanation of the influence of vegetation on methane fluxes. To date, there have been a range of research efforts focusing on this topic. King reported that the activity of methanotrophs varied among vegetation species that had different methane uptake rates (King, 1994). Kao‐Kniffin, Freyre, & Balser (2010) reported that the structure of methanogens showed no distinguishable patterns among vegetation species, and there was no correlation with methane fluxes. Another report showed that the community structures of both methanogens and methanotrophs were different between vegetation types that exhibit different methane fluxes, but the correlation between the methane‐cycling microbes and methane fluxes was not analyzed (Narihiro et al., 2011). Overall, it remains unclear how methane‐cycling microbes lead to the different methane fluxes among vegetation species. The correlation analysis between microbes and fluxes for methane will be of great significance for accurate construction of the methane‐cycling model and even prediction of global climate change.

In this study, we investigated the methanogens and methanotrophs in the Zoige wetland based on key genes involved in methane production and oxidation. The abundances were quantified by real‐time quantitative PCR, and the diversities and compositions of the microbial communities were analyzed based on the data from Illumina MiSeq sequencing. The goal of this study was to determine the aspects of methane‐cycling microbial communities that contribute to the different methane fluxes between different vegetation types in the Zoige wetland.

## 2 MATERIALS AND METHODS

### 2.1 Soil characteristics

Water‐flooded soil samples were collected in early August 2011 from two areas of soil dominated by different plant species in the Zoige National Wetland Reserve (33°56′ N, 102°52′ E): *C. muliensis* and *E. valleculosa* (Chen et al., 2011; Cui et al., 2015). A five‐point sampling method was used for each site with a sampling size of 3 ×** **3 m and soils obtained from the five‐point sampling method were mixed to represent each site (Tu et al., 2011). For each kind of sample, three sites were set and all the subsequent experiments were carried out in triplicates. The fresh soil was transported to the laboratory at 4°C for the measurement of soil physicochemical properties and methane emissions potential, and the remaining soil was stored at −20°C until required for analysis..

The moisture content (MC) was determined by drying overnight at 105°C and weighing. The pH values were measured by mixing wet‐weight soil with distilled water at a ratio of 1:1 (w/w). Organic matter (OM) was determined by the external heating‐potassium dichromate volumetric method (Tian‐Wei, 2005). The total nitrogen (TN) content was determined by the Kjeldahl method (Bremner, 1960). Ammonium nitrogen (AN) and nitrate nitrogen (NN) were determined with an AA3 continuous flow analytical system (Seal, German) (Liu, Liang, & Chu, 2017). The total phosphorus (TP) was determined by the perchloric acid‐concentrated sulfuric acid digestion and the Mo‐Sb colorimetric method (Kuo, 1996). The methane flux was measured as reported in a previous study (Yuan, Ding, Liu, Xiang, & Lin, 2014), except for modifications to the soil slurries and incubation conditions. The soil slurries contained a mixture of 20 ml fresh soil with an equal volume of sterile water. The incubation took place in the dark at 15°C for 7 days. The methane concentration was detected by Shimadzu 2010 Ultra GC‐MS (Shimadzu, Japan) with an Agilent GS‐CarbonPlot column (Agilent, USA). The injection, column and detection temperatures were 150, 35 and 200°C, respectively.

### 2.2 DNA extraction and quantitative real‐time PCR analysis

In this study, 0.5 g fresh soil from each sample was used in the DNA extraction, which was performed with a FastDNA SPIN Kit for soil (MP Biomedicals, Santa Ana, California, USA) according to the manufacturer's instructions. The DNA concentrations were measured with a Nanodrop^®^ ND‐1000 UV‐Vis spectrophotometer (Nanodrop Technologies, Wilmington, DE, USA) according to the manufacturer's directions. The DNA yield was approximately 50–350 ng μl^−1^.

Quantitative real‐time PCR (qPCR) was performed on a Bio‐Rad CFX96 connect real‐time PCR instrument (Bio‐Rad, Hercules, California, USA) with a SYBR Green qPCR kit (Takara, Dalian, Liaoning, China). The abundances of *mcrA*,* pmoA*, archaea 16S rRNA genes and bacteria 16S rRNA genes were quantified, using the primers shown in Supporting Information Table S1. The qPCR standards were generated by serial dilutions of the plasmid carrying the respective gene targets, which were the verified sequence of PCR products (MH071177 for archaea 16S rRNA genes, MH071178 for bacteria 16S rRNA genes, MH102312 for *mcrA* and MH102313 for *pmoA*) with the respective primers for the qPCR. The PCR specificity and the dimer formation of the primer were monitored by analyzing the dissociation curves. Each qPCR mixture contained 12.5 μl of 2×** **SYBR Premix ExTaq II Mix (Takara, Dalian, Liaoning, China), 1 μl of each primer (10 μmol L^−1^), 2 μl of diluted DNA (10 ng μl^−1^) and double‐distilled H_2_O to a final volume of 25 μl. All qPCRs were performed in triplicate following the PCR program consisting of 95°C of initial denaturation for 30 s, 40 cycles of 95°C for 5 s and 60°C (61°C for Archaea 16S rRNA genes) for 35 s, followed by 10 s at 95°C, and a melt curve analysis was then performed (65–95°C with increments of 0.5°C for 5 s).

### 2.3 PCR amplification and illumina MiSeq sequencing

The *mcrA* gene and *pmoA* gene were used to construct the community libraries for the methanogens and methanotrophs, respectively. The PCR primers are shown in Table 1. The PCR was performed, using Ex Taq DNA polymerase (Takara, Dalian, Liaoning, China) with 10–40 ng of template DNA in a total reaction mixture volume of 50 μl, following the Ex Taq product protocol. After purification using the AxyPrep DNA Gel Extraction Kit (Axygen Biosciences, Tewksbury, Massachusetts, USA) and quantification using a QuantiFluor™‐ST fluorometer (Promega, Madison, Wisconsin, USA), a mixture of amplicons was used for high‐throughput sequencing on an Illumina MiSeq platform with 300 bp paired‐end reads generated.

**Table 1:** Physicochemical properties of each sample

Sample	MC (w/w)	pH	OM (g kg^−1^)	TN (g kg^−1^)	TP (g kg^−1^)	AN (mg kg^‐−1^)	NN (mg kg^−1^)
Cm	0.84 **± **0.02	7.08 **± **0.00	102.18 **± **0.65^b^	1.68 **± **0.01	0.45 **± **0.00	16.53 **± **0.01	0.10 **± **0.00^a^
Ev	0.90 **± **0.03	7.1 **± **0.00^a^	91.62 **± **0.01	1.73 **± **0.00^a^	0.49 **± **0.00^b^	21.06 **± **0.03^b^	0.01 **± **0.00

*Note*. Values represent the means with standard errors. MC: moisture content; OM: organic matter; TN: total nitrogen; TP: total phosphorus; AN: ammonia nitrogen; NN: nitrate nitrogen.

a *p* ≤ 0.01.

b *p* ≤ 0.001.

### 2.4 High‐throughput sequencing data analysis

The produced paired‐end reads were assigned to samples based on their unique barcodes and saved in FASTQ format. For each sample, there were two files, fq1 and fq2, that contained the reads of each end. The FASTQ format data were first filtered to remove the low‐quality sequences (quality values < 20). Chimera sequences and short sequences were removed from the raw tags to form the clean tags used for the next analysis. Clean tags of nucleotide sequences were translated to proteins using Framebot (http://fungene.cme.msu.edu/FunGenePipeline/framebot/form.spr) to detect and correct frameshifts in the reads. After correction, sequences with ≥97% similarity were assigned to the same operational taxonomic units (OTUs) by Uclust (http://www.drive5.com/uclust/downloads1_2_22q.html) software (Edgar, 2010). For each representative sequence in each OTU, the FunGene database (http://fungene.cme.msu.edu/) (Fish et al., 2013) was used based on an RDP classifier (http://sourceforge.net/projects/rdp-classifier/) algorithm to annotate the taxonomic information (Wang, Garrity, Tiedje, & Cole, 2007; Xu, Lu, Xu, Chen, & Sun, 2016).

### 2.5 Statistical analyses

Alpha diversity was applied with QIIME 1.8.0 to analyze the complexity of species diversity of the samples (Caporaso et al., 2010) (http://qiime.org/scripts/alpha_rarefaction.html), and the results were displayed with R software (Version 2.15.3). Beta diversity on unweighted UniFrac was calculated by the QIIME software. An analysis of similarities (Anosim) provides a way to statistically test whether there is a significant difference between two or more groups of sampling units and was performed in R software with the vegan package. An independent‐samples *t*‐test was conducted to test the significance of the difference, and Pearson correlation coefficients were used to calculate the correlation in the PASW Statistics 18 (IBM, USA). Path analysis was conducted to analysis the effects of biotic and abiotic parameters on the methane emissions with using Amos 24 (IBM, USA) software (Chen et al., 2016). Before constructing a priori model of the path analysis, correlation analysis among all parameters were performed. And principle component analysis (PCA) was conducted with the PASW Statistics 18 (IBM, USA) to create an index to represent OM, TP, TN, AN and NN, which were all soil nutrients and significantly correlated with each other. The ratios of *mcrA*/*pmoA* were used as the index of microbial abundance and the observed species of methanogens and methanotrophs were used to represent microbial diversity. The parameters in the model were estimated by maximum likelihood methods and Chi‐square (χ^2^) was used to evaluate model fit (Petersen et al., 2012). Moreover, regression analyses were also conducted between methane emissions and microbial parameters and plotted in OriginPro 2017 software (OriginLab, USA).

### 2.6 Nucleotide sequence accession numbers

The nucleotide sequences found in this study were submitted to the NCBI Sequence Read Archive (SRA) database under accession numbers SRR5319771–SRR5319776 (*mcrA* gene) and SRR5319780–SRR5319785 (*pmoA* gene).

## 3 RESULTS

### 3.1 Physicochemical properties and methane flux

**Figure 1:**
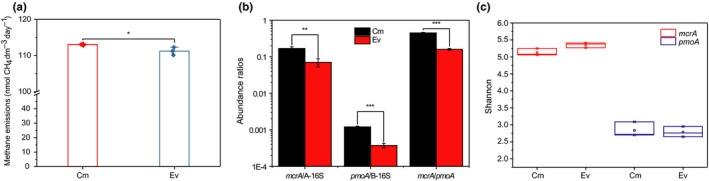
Potential rates of methane emission for soils from Cm and Ev (a), relative abundances of *mcrA* and *pmoA* genes and ratios of *mcrA*/*pmoA* for Cm and Ev samples (b), and the Shannon index of *mcrA* and *pmoA* libraries of the Cm and Ev samples (c). B‐16S, 16S rRNA of bacteria; A‐16S, 16S rRNA of archaea. The error bars indicate the standard error of the mean. * denotes *p* ≤ 0.05; ** denotes *p* ≤ 0.01; *** denotes *p* ≤ 0.001

The basic physicochemical properties of all samples are shown in Table 1. As shown, both samples had high MC and neutral pH. However, significant differences were observed between samples for all physicochemical properties except MC. The Cm samples had higher OM and NN than the Ev samples, whereas the pH, TP, TN, and AN were much higher in the Ev samples than those in the Cm samples. The methane fluxes for the Cm and Ev samples were 113.02** **±** **0.13 and 111.21** **±** **0.62 nmol CH_4_ dm^−3^ day^−1^, respectively. In addition, the potential for Cm was significantly higher than that for Ev (*p* ≤ 0.05) according to the independent‐samples *t*‐test, though the difference was small (Figure 1a).

### 3.2 Abundances of *mcrA* genes and *pmoA* genes

The gene copy numbers of *mcrA*,* pmoA*, 16S rRNA genes of bacteria (B‐16S) and 16S rRNA genes of archaea (A‐16S) in the Zoige wetland were detected (Table 2). The gene copy numbers of the *mcrA* and *pmoA* genes of per gram soil varied from the 10^6^ to 10^3^ level and the 10^6^ to 10^5^ level, respectively. The independent‐samples *t*‐test showed that Ev and Cm samples were significantly different from each other for both *mcrA* (*p* ≤ 0.001) and *pmoA* (*p* ≤ 0.05). Moreover, the abundance of both *mcrA* and *pmoA* in Cm were higher than those in Ev (Table 2). However, for the 16S rRNA genes of bacteria and 16S rRNA genes of archaea, the trends were different. The B‐16S of Cm was significantly lower than that of Ev (*p* ≤ 0.05). In addition, there was no significant difference in A‐16S between Cm and Ev samples (Table 2). The calculated relative abundances of *mcrA* and *pmoA* indicated that the ratios of *mcrA* in archaea and *pmoA* in bacteria exhibited the same features between samples as the absolute abundance of *mcrA* and *pmoA* genes (Figure 1b). Moreover, the ratio of *mcrA*/*pmoA* also showed a significantly higher value in Cm than in Ev (*p* ≤ 0.001; Figure 1b).

**Table 2:** Copy numbers of *mcrA*,* pmoA*, 16S rRNA of bacteria and 16S rRNA of archaea of the samples. The units are copies g‐soil^‐1^

Sample	*mcrA*	*pmoA*	B‐16S	A‐16S
Cm	1.80 **± **0.07 × 10^6^ b	4.03 **± **0.28 × 10^6^ a	3.34 **± **0.10 × 10^9^	1.08 **± **0.09 × 10^7^
Ev	3.99 **± **0.19 × 10^5^	2.53 **± **0.22 × 10^6^	7.09 **± **1.149 × 10^9^ a	5.96 **± **1.06 × 10^6^

*Note*. Values represent the means with standard errors. B‐16S, 16S rRNA of bacteria; A‐16S, 16S rRNA of archaea.

a *p* ≤ 0.05.

b *p* ≤ 0.001.

### 3.3 Alpha diversity of methanogens and methanotrophs

For the *mcrA* community, 528,301 sequences in the clean tags were corrected and assigned, producing 404 OTUs. For the *pmoA* community, 65 OTUs were extracted from 637,382 sequences. From the rarefaction curve based on the OTUs, we observed that the sequencing depth was sufficient to describe the community (Supporting Information Figure S1).

For alpha diversity, there were five indexes that showed microbial diversity in a single sample. The indexes were Chao1, observed species, goods coverage, phylogenetic diversity (PD whole tree) and Shannon indexes, and the values are shown in the supplementary materials (Supporting Information Tables S2 and S3). There was no significant difference in Shannon indexes between Cm and Ev samples for both methanogens and methanotrophs (Figure 1c). In addition, the other four alpha indexes also showed no significant difference between Cm and Ev.

### 3.4 Community composition based on *mcrA* and *pmoA*

**Figure 2:**
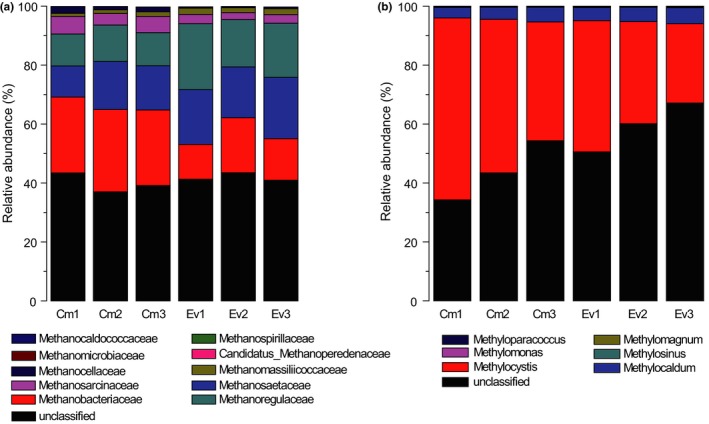
Community compositions of the *mcrA* (a) and *pmoA* (b) communities

From the relative abundance analysis of the *mcrA* community (Figure 2a), we found that Methanobacteriaceae, Methanosaetaceae, Methanoregulaceae, and Methanosarcinaceae were the dominant families in all the samples, and the relative abundance of these four dominant families in Cm were 26%, 14%, 11%, and 5%, respectively. Their relative abundances in Ev were 15%, 19%, 19%, and 3%, respectively. In addition to those four dominant families, other families were also detected at lower abundances in the soils in this study. For example, Methanomassiliicoccaceae, Methanocellaceae, and Methanomicrobiaceae had relative abundances in Cm of 1.28%, 1.71%, and 0.12%, respectively, and their relative abundances in Ev were 1.96%, 0.47%, and 0.13%, respectively. There were also three additional families with lower relative abundances. These families were *Candidatus* Methanoperedenaceae, Methanospirillaceae, and Methanocaldococcaceae.

Compared with the *mcrA* communities, the compositions of the *pmoA* communities (Figure 2b) were simple. The genus *Methylocystis* dominated all samples in the Zoige wetland, with abundances of 51% and 35% for Cm and Ev, respectively. In addition to *Methylocystis*,* Methylocaldum* was also an important genus in both Cm and Ev with abundances of approximately 4% and 5%, respectively. Other genera with relative abundances less than 1% were also found in these samples, such as *Methylosinus*,* Methylomonas*,* Methylomagnum*, and *Methyloparacoccus*.

### 3.5 Beta diversity of methanogens and methanotrophs

**Figure 3:**
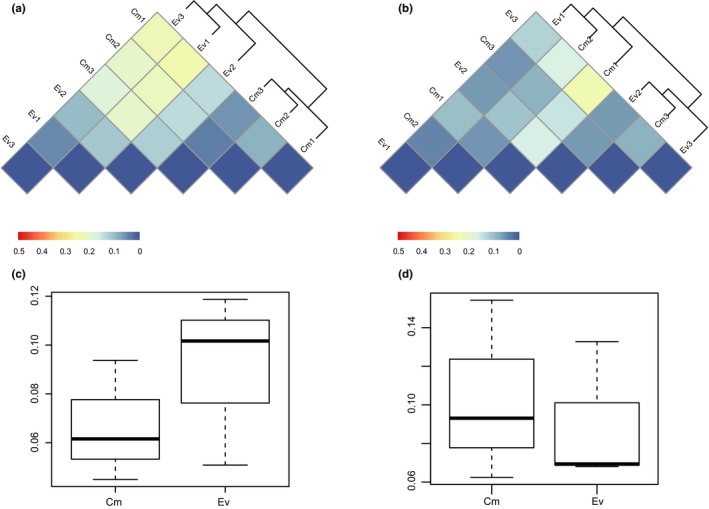
Heatmap of weighted UniFrac distances for *mcrA* (a) and *pmoA* (b) communities and boxplot of beta diversities for *mcrA* (c) and *pmoA* (d)

The weighted UniFrac distance was used to compare the methanogen and methanotroph communities among samples. For methanogens, the heatmap of weighted UniFrac distances among samples showed that samples were obviously grouped into two clusters according to vegetation type (Figure 3a). The Anosim results showed an *R* value of 0.48, which indicates that the difference between Cm and Ev groups is greater than that within groups. However, the *p* value of 0.20 indicates that this difference between Cm and Ev was not significant. Moreover, the boxplot (Figure 3c) shows the beta diversities of Cm and Ev more intuitively, and no significant difference was found between Cm and Ev by a *t*‐test.

For methanotrophs, the heatmap of weighted UniFrac distances shows that samples from Cm and Ev were clustered together (Figure 3b). The Anosim indicated no significant difference between Cm and Ev (*p* > 0.05). Moreover, the boxplot (Figure 3d) clearly shows that Cm and Ev had similar beta diversities. No significant difference was found between Cm and Ev according to a *t*‐test.

### 3.6 Correlation analysis

**Figure 4:**
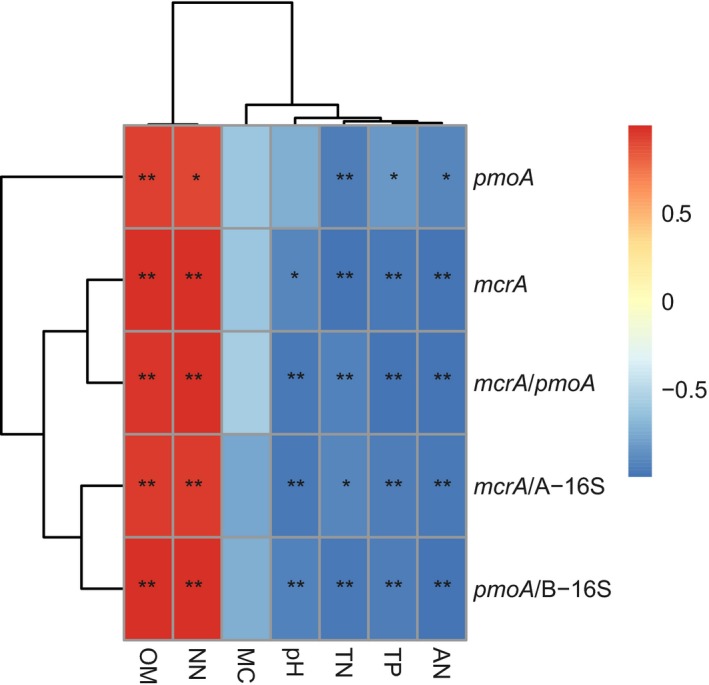
Heatmap of the Pearson correlation coefficients between physicochemical properties and abundances or abundance ratios. MC, moisture content; OM, organic matter; TP, total phosphorus; TN, total nitrogen; NN, nitrate nitrogen; AN, ammonia nitrogen. * denotes *p* ≤ 0.05; ** denotes *p* ≤ 0.01

Of the abundance and composition of both methanogens and methanotrophs, only the abundance showed a significant difference between samples. Therefore, a correlation analysis between environmental factors and microbial abundance was carried out to try to explain the differences in abundance between samples (Figure 4). The result showed that the absolute or relative abundance of *mcrA* and *pmoA* and even the ratio of *mcrA/pmoA* were all positively correlated with OM and NN and negatively correlated with pH, TP, TN, and AN (except for the absolute abundance of *pmoA* with pH) (Figure 4). In addition, the correlation coefficient between the *mcrA/pmoA* ratios and the methane production potentials was calculated to be 0.816 and was significant at the 0.05 level.

Although there were no significant differences in the microbial community between Cm and Ev, the absolute abundance of each methanogen or methanotroph would be varied between Cm and Ev due to the significant difference in community abundance. Then, the correlation between absolute abundance of each methanogen or methanotroph and soil physicochemical properties was calculated (Supporting Information Figure S2). Similar to the community abundance, most of the methanogens showed strong correlations with soil physicochemical properties. To be specific, the four dominant families of methanogens showed significant positive correlations with OM and NN and significant negative correlations with TP, TN, and AN (Supporting Information Figure S2a). Compared to methanogens, less methanotrophs showed correlations with soil physicochemical properties (Supporting Information Figure S2b). The dominant methanotroph, *Methylocystis*, showed significant positive correlations with OM and NN and negative correlations with AN, TN, TP, and pH.

**Figure 5:**
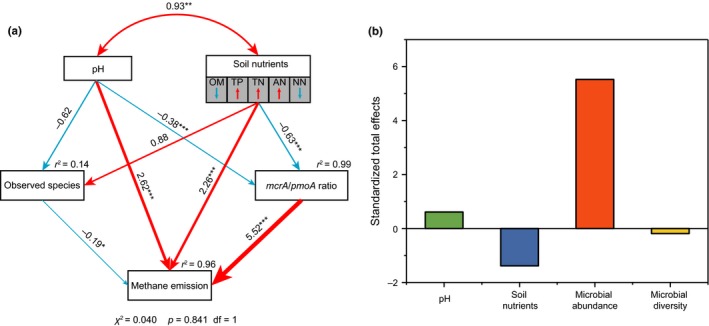
The model of path analysis to examining multivariate effects on methane (a) and standardized total effects of pH, soil nutrients, microbial abundance and microbial diversity on methane (b). The total observed species of methanogens and methanotrophs were used to represent microbial diversity and *mcrA*/*pmoA* ratios were used to represent microbial abundance. The color of arrow means positive (red) or negative (blue) relationships. The width of arrow is proportional to the strength of the relationship. Double‐layer rectangles represent the principle component 1 from the PCA conducted for soil nutrients. Soil nutrients includes organic matter (OM), total phosphorus (TP), total nitrogen (TN), ammonia nitrogen (AN) and nitrate nitrogen (NN). The red symbol “↑” and blue symbol “↓” indicate a positive or negative relationship between the variables and the principle component 1 from the PCA, respectively. The corresponding numbers adjacent to arrows are standardized path coefficients, which reflect the effect size of the relationship. The proportion of variance explained (*r* ^2^) are showed alongside each response variables in the model. Goodness‐of‐fit statistics for the model are shown below the model. *p ≤ 0.05, **p ≤ 0.01, ***p ≤ 0.001

**Figure 6:**
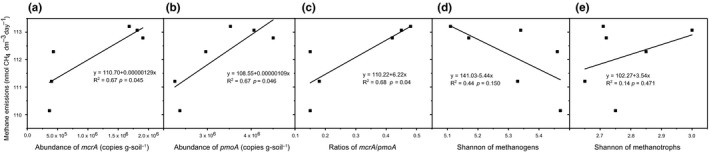
Relationships between methane emissions and the microbial parameters. The microbial parameters include the abundance of methanogens (a), the abundance of methanotrophs (b), the ratio of methanogens to methanotrophs (c), the diversity of methanogens (d) and the diversity of methanotrophs (e)

A path analysis was conducted to determine the factors responsible for the potential methane emissions (Figure 5a). Taking into account the relevance of environmental factors, PCA was conducted to create a multivariate functional index for the physicochemical factors of soil nutrients, which were all significantly correlated (Supporting Information Table S4) (Chen et al., 2016). Principal component 1 (PC1), which explained 97.61% of the total variance (Supporting Information Table S5), was then introduced as the index of soil nutrients into the path analysis. In addition to the soil nutrients, the pH also showed a significant correlation with microbial abundance, but MC showed no significant correlations with either microbial abundance or the methane emissions (Supporting Information Table S4). Hence, PC1 of soil nutrients and pH included in the construction of an *a priori* model in this study. The abundance and diversity were included in the model as microbial parameters. In the test of goodness‐of‐fit for the model, the χ^2^ was 0.040 with *p* > 0.05 (Figure 5a), which indicates good fit of the model (the model has a good fit when χ^2^ is low (~≤2) and is high (traditionally > 0.05)) (Schermellehengel, Moosbrugger, & MüLLER, 2003). The model explained 96% of the variance in the methane emissions. Microbial abundance had the highest direct positive effect on methane emissions followed by soil nutrients and pH, and microbial diversity had a direct negative effect on methane emissions. After taking all the direct and indirect effects on methane emissions into account, the standardized total effects on methane were calculated (Figure 5b). The result showed that the microbial abundance was the most important factor influencing methane emissions with much greater effects than microbial diversity, pH and soil nutrients. Moreover, the regression analysis between methane emissions and microbial parameters also showed that, methane emissions significantly increased with the increase in the abundance of methanogens and methanotrophs and the ratio of methanogens to methanotrophs, but not with the diversities of methanogens and methanotrophs (Figure 6).

## 4 DISCUSSION

Methane flux, which is controlled by the belowground methane‐cycling microbes through producing and oxidizing methane (Aronson et al., 2013), varies among vegetation species (King, 1994; Ström et al., 2005). In the current study samples of soils dominated by two different vegetation species, which were reported to have different methane fluxes in the field (Chen et al., 2009), were taken to analyze the methane‐cycling microbes and their correlations with soil physicochemical properties and methane flux.

For both methanogens and methanotrophs, Cm and Ev showed significant differences in not only the absolute abundance but also the relative abundances vs. total archaea or bacteria. This result indicated that the difference in the absolute abundances of methanogens and methanotrophs between the two sites were not caused by differences in the total abundances of bacteria and archaea. This specific difference in the functional microbes between *C. muliensis* and *E. valleculosa* may be correlated with the different methane fluxes, which is the result of the balance between methane production and methane consumption and has been reported to be different between *C. muliensis* and *E. valleculosa* in the Zoige wetland (Chen et al., 2009). In addition, previous reports have shown that the *mcrA/pmoA* ratio is correlated with methane fluxes (Chaudhary, Kim, & Kang, 2018; Liu, Wu, et al., 2017). Therefore, the *mcrA*/*pmoA* ratios were also calculated in this study to determine the correlation with methane flux. The *mcrA*/*pmoA* ratios were higher under Cm than under Ev. The methane fluxes measured in this study also showed a higher value for Cm than Ev (Figure 1a), which is consistent with previous reports (Chen et al., 2009; Chen, Yao et al., 2008). In addition, the result of the Pearson correlation coefficient analysis reflected a significant correlation between the *mcrA/pmoA* ratios and methane production potential and also the regression analysis results showed the same relationship (Figure 6c). The methane emissions also significantly increased with the increase in individual abundances of *mcrA* and *pmoA* (Figure 6a and b). And the path analysis also showed a positive effect of abundance on methane emissions (Figure 5a and b). All these results suggest that the difference in the methane‐cycling microbial abundance contributes to the methane flux differences.

However, unlike the abundance, the diversity of the *mcrA* and *pmoA* communities did not vary with vegetation species. Moreover, the composition of methanotroph communities between Cm and Ev were similar, and no significant difference was found between Cm and Ev in terms of the composition of methanogen communities. It has been stated that the same family of plants tends to have similar rhizosphere microbial community compositions (Du, Xie, Cai, Tang, & Guo, 2016). In addition, in this study, Cm and Ev are two different genera in the same family, Cyperaceae. Hence, the relative distance in taxonomy of the vegetation species may explain the similarities in the diversity and composition of the microbial communities. The similarities in this study are not an exception in related studies, although the methane flux performances of Cm and Ev differed. Other studies have also reported similar compositions of methanogens across samples that had different methane emission rates (Juottonen, Tuittila, Juutinen, Fritze, & Yrjälä, 2008; Ramakrishnan, Lueders, Dunfield, Conrad, & Friedrich, 2001). In addition, Kao‐Kniffin et al. (2010) reported that methanogen communities did not affect methane fluxes. Moreover, among all the environmental factors and microbial parameters, the microbial diversity had the least effect on methane emissions (Figure 5a and b). The regression analysis also showed that methane emissions did not significantly vary with the variance of the diversities of both methanogens and methanotrophs (Figure 6d and e). These results suggest that in this study, the compositions of the methane‐cycling communities had no significant contributions to the methane flux difference between vegetation types.

Although no significant differences were observed in methanogen and methanotroph community compositions, the dominant families or genera in Cm were more abundant in absolute terms than those in Ev due to the higher abundances of total methanogens and methanotrophs in Cm. The dominant families of methanogens in this study were Methanobacteriaceae, Methanosaetaceae, Methanoregulaceae, and Methanosarcinaceae, which are all common families in peatlands (Basiliko et al. 2003; Kotsyurbenko et al. 2007). As the Zoige wetland is also a peatland with large carbon stocks, the dominance of these families is logical. The prevalence of these families in peatlands may indicate that these families prefer environments with abundant OM, which is consistent with the observations in this study (Supporting Information Figure S2). This result explains the high absolute abundance of these families in Cm, which had a higher OM content than Ev. Moreover, Methanobacteriaceae and Methanosarcina both have wide ranges of substrate utilization for methane production. The former exhibits both methylotrophic and hydrogenotrophic activity (Nazaries, Murrell, Millard, Baggs, & Singh, 2013), and the latter exhibited acetotrophic, methylotrophic, and hydrogenotrophic activity for methanogenesis (Ferry, 2010). Chin et al. (2004) showed that the suppression of high phosphate to Methanosarcinaceae, which may be related to its high absolute abundance in Cm in this study, as the TP content was lower in Cm than Ev. This property could also explain the negative correlation of Methanosarcinaceae with TP (Supporting Information Figure S2). Methanosaetaceae was suggested to be the representative family of methanogens for acetotrophic methanogenesis (Kruger, Frenzel, Kemnitz, & Conrad, 2005), and Methanoregulaceae was a new family of hydrogenotrophic methanogens (Brauer, Cadillo‐Quiroz, Ward, Yavitt, & Zinder, 2011). These dominant families revealed the versatility and diversity of substrate utilization for methane production in the Zoige wetland. In addition, unlike the studies that reported that Methanoregulaceae is always found in acidic environments (Sakai et al., 2012; Sun, Brauer, Cadillo Quiroz, Zinder, & Yavitt, 2012), this study represents the first time that this family has been detected in a neutral environment. The methanotrophs in our study exhibited lower diversity than methanogens. This result is realistic for wetlands, as methanogens thrive on the organic substrates provided by the vegetation in anaerobic conditions, and methanotrophs are limited by the available oxygen (Conrad, 2004). The dominant genus of methanotrophs was *Methylocystis* in all the samples from the Zoige wetland. As a ubiquitous genus of aerobic methanotrophs in many ecosystems (Chen, Dumont, Cébron, & Murrell, 2007; Kip et al., 2010), *Methylocystis* has been shown to be particularly rich in peat soils (Chen, Dumont, et al., 2008; Dedysh, 2009; McDonald, Uchiyama, Kambe, Yagi, & Murrell, 1997). This result explains not only the dominance of *Methylocystis* in the Zoige wetland but also the higher absolute abundance of *Methylocystis* in Cm than Ev, as there was a higher OM content in the Cm samples than in the Ev samples. Moreover, the occurrence of the genus *Methylocaldum* in all the samples from the Zoige wetland is interesting, as most of the strains of this genus are thermophilic or thermotolerant (Nazaries et al., 2013), and the Zoige wetland is an alpine wetland. However, in this study, most of the strains in this genus are unclassified species, which may indicate new species of *Methylocaldum* adapted to low temperature and explain its existence in the Zoige wetland, but more detailed studies are required to confirm a new species.

In the Zoige wetland, Cm and Ev samples, methanogens and methanotrophs were both significantly different in abundance only. In addition, the varied abundances were correlated with some of the soil physicochemical properties. The OM and NN seem to promote the growth of both methanogens and methanotrophs, as they were positively correlated with all abundances (Figure 4) and have positive effects on microbial abundance (Figure 5a). The positive correlation with OM can be easily understood, as the decomposition of OM provides energy and nutrients for microbial growth (Fontaine, Mariotti, & Abbadie, 2003; Refai, Wassmann, & Deppenmeier, 2014). It has been shown that the abundance of methanogens are significantly increased by OM addition (Asakawa, Akagawa‐Matsushita, Koga, & Hayano, 1998). Moreover, NN can also promote microbial growth through assimilation into organic nitrogen (Rice & Tiedje, 1989), which has been reported to increase microbial biomass (Treseder, 2008). The AN was previously reported to inhibit methanogens (Shrestha, Shrestha, Frenzel, & Conrad, 2010) and methanotrophs (Nyerges & Stein, 2009) even when concentrations were at the micromole level, such as 300 μM for methanotrophs (Kim, Veraart, Meima‐Franke, & Bodelier, 2015; Van Der Nat, de Brouwer, Middelburg, & Laanbroek, 1997), which is much lower than the AN concentration in this study. In addition, TN was also negatively related to the abundance of methanogens and methanotrophs (Figure 4 and Figure 5a), which may be partly associated with the inhibition of AN. Most known methanogens and methanotrophs grow optimally at near‐neutral pH (Horn, Matthies, Küsel, Schramm, & Drake, 2003; McDonald, Radajewski, & Murrell, 2005), which could help to explain the prevalence of methanogens and methanotrophs in the Zoige wetland that had a neutral pH in our study. Furthermore, a higher pH than neutral was reported to be correlated with a reduction in methane production (Barredo & Evison, 1991). This result is consistent with the negative correlations between microbial abundances and pH in our study (Figure 4 and Figure 5a). In addition, TP was negatively correlated with the microbial abundances, which is similar to the previously reported inhibition of methanotrophs (Alam, Xia, & Jia, 2014). These correlations with physicochemical properties can help to explain the differences in the abundances of methanogens and methanotrophs.

In conclusion, in soil samples of vegetation types with small but significant differences in methane fluxes, both methanogens and methanotrophs were significantly different in abundance but similar in diversity and composition. The abundances of methanogens and methanotrophs in the Cm soil were significantly higher than those in Ev soil, and the potential methane emissions significantly increased with the increase in abundances at the two sampling sites. However, no significant differences were found in the diversity and composition of methanogens and methanotrophs between the two sampling sites. The differences in abundance were inferred to be related to the differences in environmental factors according to the correlation analysis. Moreover, in the analysis of interactions among the soil parameters, microbial parameters and methane, the microbial abundance had the strongest impact on methane, which far exceeded those of microbial diversity or the measured soil properties. In addition, methane emissions significantly varied with the variance of abundance of both methanogens and methanotrophs but not significantly varied with the variance of the diversities of both methanogens and methanotrophs. These results therefore indicate that the abundance rather than the composition of methane‐cycling microbes is the main contributor to the observed differences in the methane fluxes between vegetation types in the Zoige wetland. This conclusion contributes to explaining the different methane fluxes among vegetation species under global warming.

## ACKNOWLEDGMENTS

This work was supported by National Key Program of China (2016YFC0502104), National Natural Science Foundation of China (41671270, 41001151), Key Research Program of the Chinese Academy of Sciences (ZDRW‐ZS20165) and Youth Innovation Promotion Association CAS (2016039). We thank Prof. Robert Gunsalus for the helpful discussion.

## COMPLIANCE WITH ETHICAL STANDARDS

This article does not contain any studies with human participants or animals performed by any of the authors.

## CONFLICT OF INTEREST

The authors declare that they have no conflict of interest.

## DATA ACCESSIBILITY STATEMENT

The nucleotide sequences found in this study were submitted to the NCBI Sequence Read Archive (SRA) database under accession numbers SRR5319771–SRR5319776 (*mcrA* gene) and SRR5319780–SRR5319785 (*pmoA* gene).
