# Supplementary material for: Abundance, rather than composition, of methane‐cycling microbes mainly affects methane emissions from different vegetation soils in the Zoige alpine wetland
Source: Microbiologyopen. 2018 Jul 26;8(4):e00699. doi: 10.1002/mbo3.699 (PMC6460274; doi:10.1002/mbo3.699)
Supplement: Supplementary file 1 [file MBO3-8-e00699-s001.pdf]

**Supplementary Material**

**MicrobiologyOpen**

# Abundance, rather than composition, of methane-cycling microbes mainly affects methane emissions from different vegetation soils in the Zoige alpine wetland

**Yanfen Zhang[a, b], Mengmeng Cui[c, b], Jingbo Duan[d, b], Xuliang Zhuang[a,b], Guoqiang Zhuang[a,b], Anzhou Ma[a,b]**

[a] Key Laboratory of Environmental Biotechnology, Research Center for Eco-Environmental Sciences, Chinese Academy of Sciences, Beijing 100085, China

[b] University of Chinese Academy of Sciences, Beijing 100049, China

[c] National Laboratory of Biomacromolecules, Institute of Biophysics, Chinese Academy of Sciences, Beijing 100101, China

[d] Key Laboratory of Environmental Optics & Technology, Anhui Institute of Optics and Fine Mechanics, Chinese Academy of Sciences, Hefei 230031, China

Corresponding author: azma@rcees.ac.cn, gqzhuang@rcees.ac.cn +86 010 6292 3562

**Table S1** Primers used in this study.

| Primer | Application | Specificity | Sequence (5'-3') | References |
|---|---|---|---|---|
| MLf | qPCR and Miseq sequencing | *mcrA* | GGTGGTGTMGGATTCACACARTAYGCWACAGC | (Luton et al., 2002) |
| MLr | qPCR and Miseq sequencing | *mcrA* | TTCATTGCRTAGTTWGGRTAGTT | |
| A189 | qPCR and Miseq sequencing | *pmoA* | GGNGACTGGGACTTCTGG | (Kolb et al., 2003) |
| mb661 | qPCR and Miseq sequencing | *pmoA* | CCGGMGCAACGTCYTTACC | |
| A349F | qPCR | Archaea 16S rRNA | GYGCASCAGKCGMGAAW | (Frank et al., 2013) |
| A806R | qPCR | Archaea 16S rRNA | GGACTACVSGGGTATCTAAT | |
| 515F | qPCR | Bacteria 16S rRNA | GTGCCAGCMGCCGCGGTAA | (Evans et al., 2014) |
| 806R | qPCR | Bacteria 16S rRNA | GGACTACHVGGGTWTCTAAT | |

**Table S2** Alpha diversity indexes of *mcrA* libraries in the three kinds of samples

| SampleID | chao1 | goods_coverage | observed_species | PD_whole_tree | shannon |
|---|---|---|---|---|---|
| Cm | 345±4 | 0.9943±0.0001 | 285±7 | 15.88±0.27 | 5.21±0.07 |
| Ev | 350±24 | 0.9949±0.0005 | 291±4 | 16.12±0.27 | 5.42±0.05 |

Values represent means with standard errors.

**Table S3** Alpha diversity indexes of *pmoA* libraries in the three kinds of samples

| SampleID | chao1 | goods_coverage | observed_species | PD_whole_tree | shannon |
|---|---|---|---|---|---|
| Cm | 58±2 | 0.9997±0.0001 | 58±1 | 3.39±0.19 | 2.81±0.10 |
| Ev | 57±2 | 0.9998±0.0001 | 56±2 | 3.49±0.20 | 2.75±0.06 |

Values represent means with standard errors.

**Table S4** Pearson correlation coefficient among the variables in this study

| MC | pH | OM | TN | TP | AN | NN | methane | diversity | |
|---|---|---|---|---|---|---|---|---|---|
| -0.565 | -0.969** | 0.972** | -0.934** | -0.985** | -0.993** | 0.988** | 0.816* | -0.260 | abundance |
| | 0.602 | -0.633 | 0.554 | 0.635 | 0.601 | -0.589 | -0.236 | -0.065 | MC |
| | | -0.912* | 0.853* | 0.954** | 0.944** | -0.929** | -0.679 | 0.198 | pH |
| | | | 0.986** | -0.951** | -0.992** | 0.995** | 0.799 | -0.335 | OM |
| | | | | .891* | 0.964** | -0.974** | -0.797 | 0.439 | TN |
| | | | | | 0.979** | -0.970** | -0.801 | 0.124 | TP |
| | | | | | | -0.999** | -0.823* | 0.285 | AN |
| | | | | | | | 0.834* | -0.314 | NN |
| | | | | | | | | -0.423 | methane |

MC, moisture content; OM, organic matter; TP, total phosphorus; TN, total nitrogen; NN, nitrate nitrogen; AN, ammonia nitrogen. * denotes $P \leq 0.05$; ** denotes $P \leq 0.01$

**Table S5** Results of the principal components analysis (PCA) of soil nutrients

| **Variable** | **PC1** |
|---|---|
| Organic matter | -0.997 |
| Total nitrogen | 0.975 |
| Total phosphorus | 0.970 |
| Ammonium nitrogen | 0.999 |
| Nitrate nitrogen | -1.000 |
| **Cumulative (%)** | **97.611** |

**Figure S1** Rarefaction curves of *mcrA*(a ) and *pmoA*(b ) libraries.

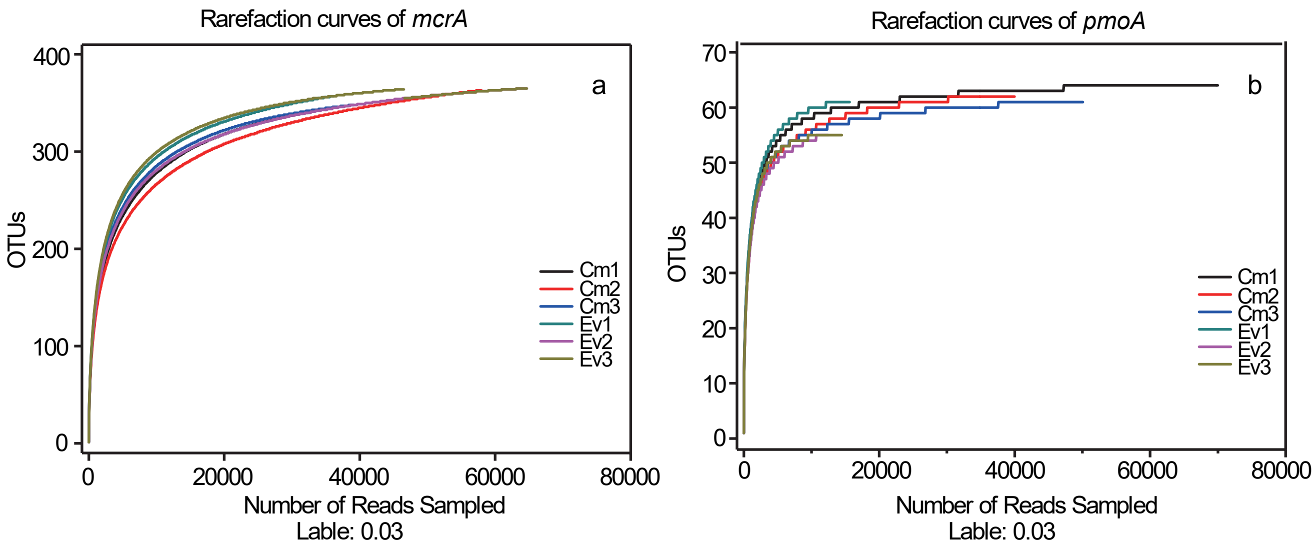

**Figure S2** Correlation analysis of physicochemical parameters with compositions of methanogens (a) and methanotrophs (b). MC, moisture content; OM, organic matter; TP, total phosphorus; TN, total nitrogen; NN, nitrate nitrogen; AN, ammonia nitrogen. * denotes P ≤ 0.05; ** denotes P ≤ 0.01

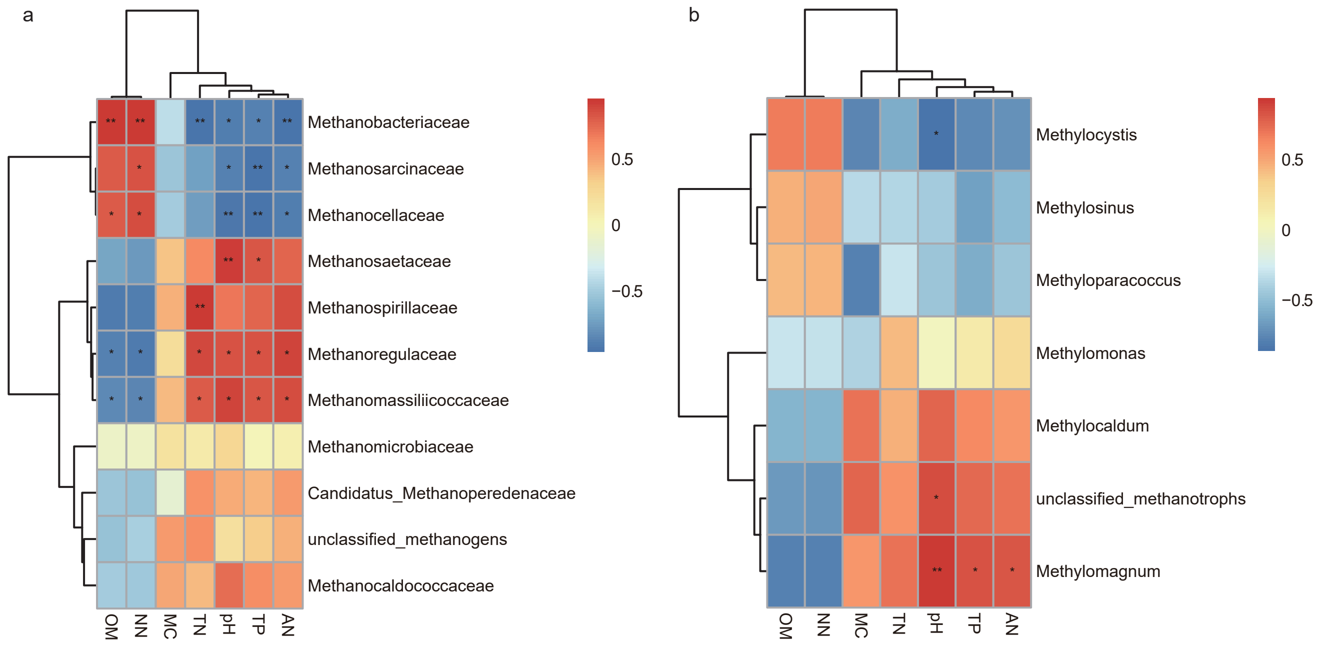


## References


Evans, C. C., Lepard, K. J., Kwak, J. W., Stancukas, M. C., Laskowski, S., Dougherty, J., Moulton, L., Glawe, A., Wang, Y., Leone, V., Antonopoulos, D. A., Smith, D., Chang, E. B. & Ciancio, M. J. 2014. Exercise Prevents Weight Gain and Alters the Gut Microbiota in a Mouse Model of High Fat Diet-Induced Obesity. *PLoS ONE,* 9**,** e92193.

Frank, K. L., Rogers, D. R., Olins, H. C., Vidoudez, C. & Girguis, P. R. 2013. Characterizing the distribution and rates of microbial sulfate reduction at Middle Valley hydrothermal vents. *The ISME Journal,* 7**,** 1391-1401.

Kolb, S., Knief, C., Stubner, S. & Conrad, R. 2003. Quantitative detection of methanotrophs in soil by novel pmoA-targeted real-time PCR assays. *Applied and Environmental Microbiology,* 69**,** 2423-2429.

Luton, P. E., Wayne, J. M., Sharp, R. J. & Riley, P. W. 2002. The mcrA gene as an alternative to 16S rRNA in the phylogenetic analysis of methanogen populations in landfill. *Microbiology-Sgm,* 148**,** 3521-3530.
